# Vulvar endometriosis presenting as a periurethral mass: a rare case report and a brief literature review

**DOI:** 10.1097/RC9.0000000000001032

**Published:** 2026-09-25

**Authors:** Maryam Deldar Pasikhani, Sara Valipour, Zeinab Mansouri, Ehsaneh Khodayar, Niloofar Hoorshad

**Affiliations:** a Division of Female Pelvic Medicine and Reconstructive Surgery, Department of Obstetrics and Gynecology, Imam Khomeini Hospital Complex, Tehran University of Medical Sciences, Tehran, Iran; b Department of Obstetrics and Gynecology, Imam Khomeini Hospital Complex, Tehran University of Medical Sciences, Tehran, Iran

**Keywords:** case report, complete excision, endometriosis, perineum, vulva

## Abstract

**Introduction:**

Endometriosis is a condition characterized by the presence of endometrial-like tissue outside its normal uterine location. While the pelvic cavity is the most common site of involvement, endometriosis can also affect other areas, with vulvar endometriosis being rare.

**Presentation of case:**

This case report describes a 44-year-old woman with a history of unilateral swelling of the upper part of the labia majora, accompanied by deviation of the urethral meatus, which began 5 years earlier following a spontaneous vaginal delivery. The lesion was excised completely, and based on pathological findings, vulvar endometriosis was diagnosed.

**Discussion:**

The unusual location and atypical appearance of the lesion set this case apart from previously reported cases of vulvar endometriosis. Reporting this case may assist clinicians in recognizing similar presentations and reducing the likelihood of misdiagnosis.

**Conclusion:**

Vulvar endometriosis is a rare entity with heterogeneous clinical manifestations. Accurate diagnosis relies on meticulous evaluation and histopathological confirmation. Treatment is by complete surgical excision, offering symptomatic relief and minimizing the risk of recurrence.

## Introduction

The endometrium is the innermost layer of the uterus. The presence of endometrial-like tissue at any site outside the uterine cavity is defined as endometriosis^[^1,2^]^.

It is known as a hormone-dependent, chronic inflammatory disease. Approximately 10% of reproductive-age females are affected by endometriosis; however, the prevalence in the general population cannot be definitively determined due to asymptomatic cases^[^3–5^]^.

HIGHLIGHTS

- Perineal endometriosis is a rare form of extra-pelvic endometriosis with highly variable and atypical clinical presentations.
- Imaging studies are non-specific, making clinical suspicion and differential diagnosis essential.
- Histopathological examination remains the gold standard for definitive diagnosis.
- Complete surgical excision with clear margins is the treatment of choice.
- Perineal endometriosis should be considered in women with cyclic perineal pain and a history of vaginal delivery.

Based on the location of ectopic endometrial implants, endometriosis is classified into intrapelvic and extra-pelvic forms. However, it commonly affects the pelvic cavity^[^6^]^, including the ovaries, fallopian tubes, and the uterosacral ligaments posterior to the uterus. Extra pelvic endometriosis can also occur in the perineum, urinary system, gastrointestinal tract, nervous system, chest, subcutaneous tissue, and skin^[^7^]^. Extra pelvic endometriosis represents a small subset of cases, reported in up to 12% of patients with endometriosis^[^1,8,9^]^.

Vulvar endometriosis is rare, affecting up to 1% of patients, and may involve the vulvar or perineal skin, subcutaneous tissue, or the perianal sphincteric muscles^[^10,11^]^. It can occur spontaneously or as a secondary condition following vulvovaginal surgery, episiotomy, or obstetric lacerations, even when these wounds are adequately repaired^[^12^]^.

Lesions of vulvar endometriosis differ from those typically observed within the pelvic cavity. Vulvar endometriosis can present as ulcers, nodules, masses or swellings, bluish cutaneous discoloration, and polypoid lesions^[^12^]^.

This report describes a patient presenting with unilateral swelling of the labia majora, situated away from the episiotomy scar, accompanied by deviation of the urethral meatus.

This study has been reported in line with the SCARE 2025 criteria^[^13^]^.

## Case presentation

A 44-year-old woman, gravida 4, para 2, abortion 2 (G4P2A2), with a regular menstrual history, presented with unilateral swelling of the right upper part of the vulva between the labia majora and minora. She reported no prior medical illnesses or surgical history. The swelling began 5 years ago following a vaginal delivery that included a right mediolateral episiotomy, which was remote from the site of the subsequent induration. There was no evidence of any other perineal trauma or surgery. Approximately 6 months before presenting to our clinic, she underwent an incision and drainage procedure, and the mass was found to contain a thick mixture of blood and fluid. Since then, the mass has progressively enlarged. The patient reported cyclic perineal pain (scored 6 out of 10 on a scale where 10 indicates the worst pain), lasting for 10 days each month, accompanied by a palpable mass in the right upper region of the vulva; however, there was no evidence of discharge or warmth at the site. The patient noted that the mass had progressively enlarged, causing difficulty with urination and sexual intercourse.

The perineal examination revealed unilateral swelling at the right upper portion of the labia majora, distant from the site of the previous episiotomy scar. Medially, it was in close proximity to the right labia minora and partially displaced the urethra due to compression, and, superiorly, the lesion was confined to the pubic region. Inferiorly, there was no involvement of the anal region. A palpable, semi-mobile, round mass measuring approximately 6 cm in diameter, soft in consistency, was noted without any lymphadenopathy or overlying skin changes. It was irreducible and did not increase in size with coughing or the Valsalva maneuver, making an inguinal hernia less likely. Figures 1 (a) and (b) show the vulvar region before and after surgery.

**Figure 1.**
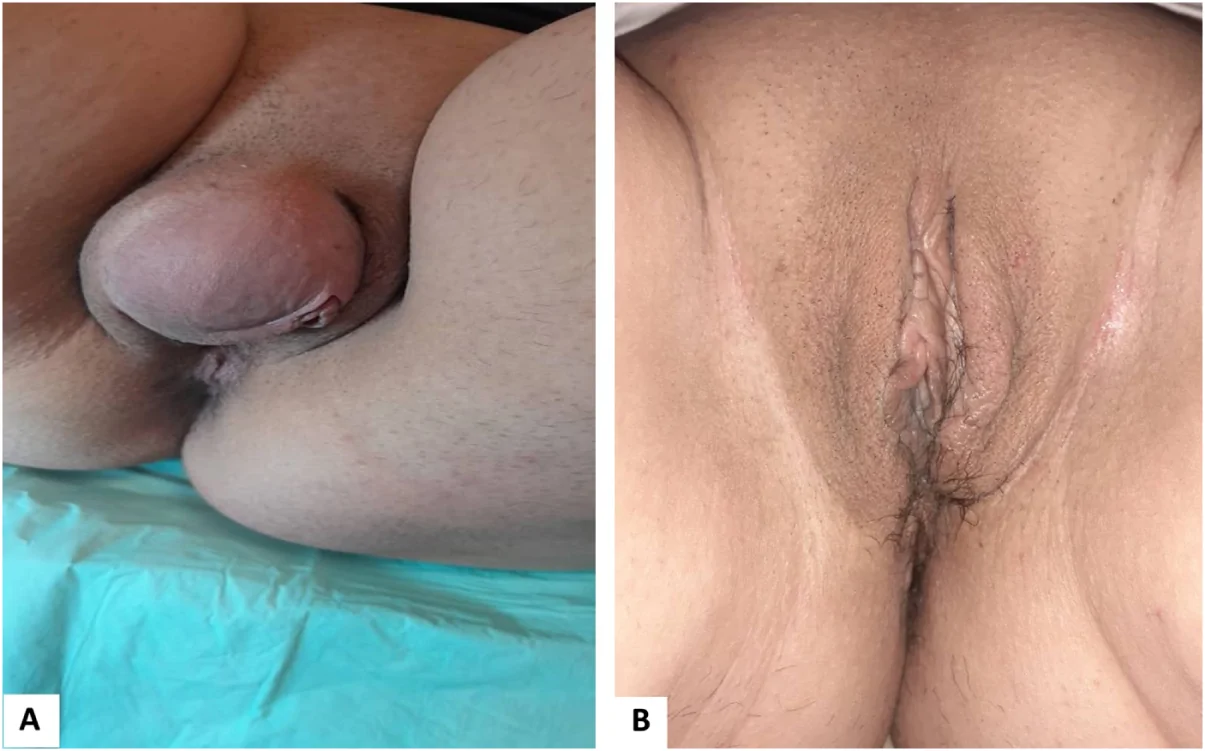
The vulvar region: (A) before mass excision; (B) after mass excision and healing of the surgical site.

Trans perineal ultrasound findings demonstrated a well-circumscribed cystic lesion with homogeneous, low-level internal echoes, reflecting chronic hemorrhagic contents. The lesion was located anterior to the pubic symphysis, measuring approximately 65 × 55 mm. It did not exhibit the typical characteristics of a Bartholin’s cyst, which is typically located in the posterolateral lower vestibule, nor those of a Skene duct cyst, which usually arises adjacent to the distal urethra. Urethral diverticulum was considered less likely because the lesion lacked communication with the urethra and was located predominantly superior rather than circumferential to the urethral tract. Furthermore, due to the absence of erythema or tenderness, a vulvar abscess was considered less likely.

Regarding the patient’s complaints (cyclic pain and a history of the same lesion in the same anatomic location), clinical examination (a well-defined, mobile, non-tender mass), and imaging findings (not indicating a vulvar abscess), we decided not to perform an MRI due to limited resources; instead, an examination under general anesthesia followed by surgical excision of the lesion was planned. As we estimated a blood loss of about 300 cc during surgery, one unit of packed red blood cells was reserved preoperatively. One gram of cefazolin was administered 30 minutes before surgery, and the patient was positioned in the lithotomy position. A 4 cm incision was made at the most prominent part of the cyst. The cyst was then sharply dissected using Metzenbaum scissors. A metal catheter was placed in the urethra and used as a guide to prevent urethral injury [Fig. 2 (a)]. The cyst was filled with a thick, chocolate-colored fluid. Complete excision was ensured by circumferential dissection of the cyst wall without gross residual tissue.

**Figure 2.**
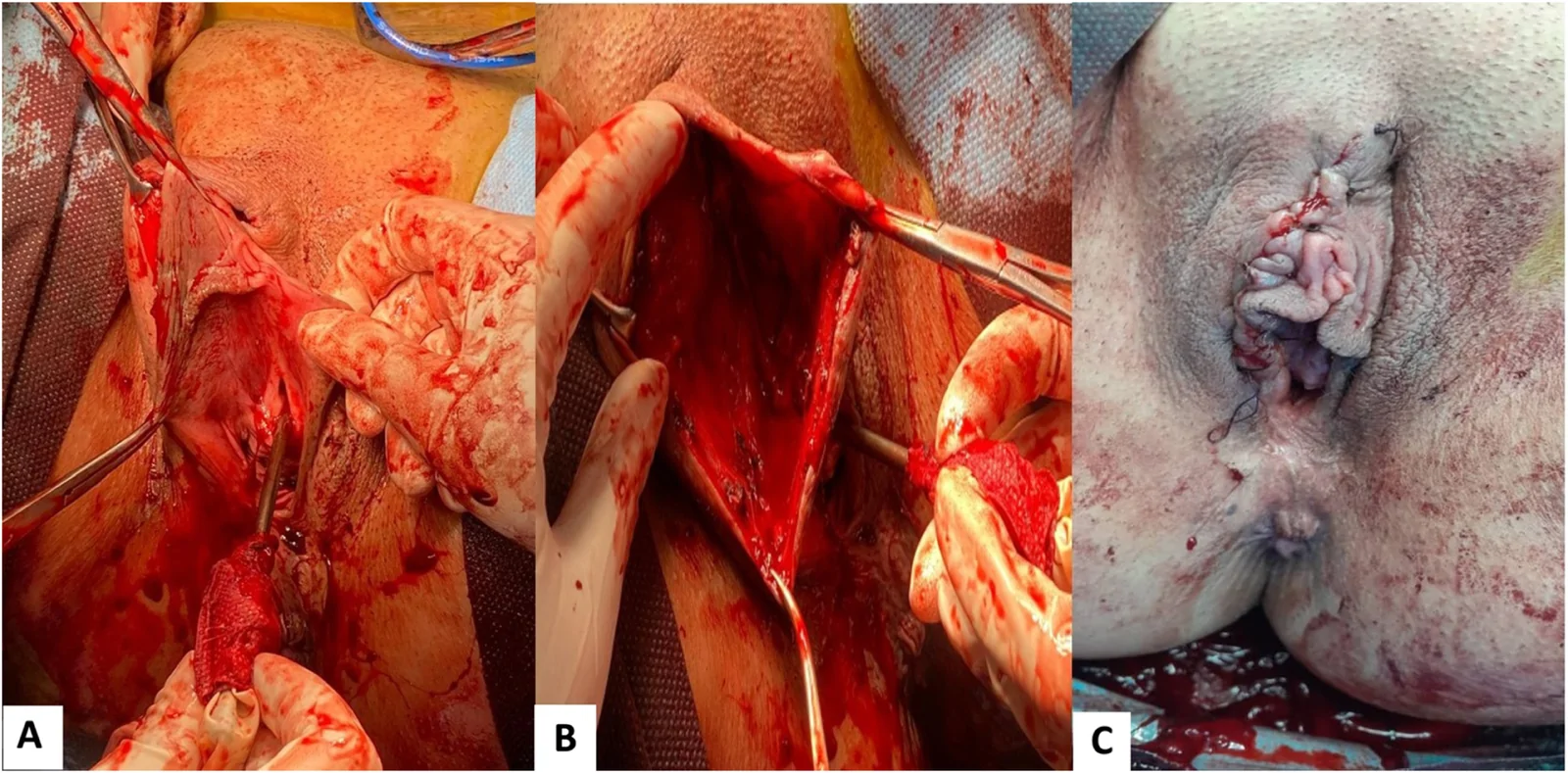
The process of surgery: (A) a metal catheter was used as a guide; (B) the surgical bed after mass excision; and (C) after completion of surgery.

After cyst excision, the surrounding skin was trimmed, and the surgical bed was repaired with a continuous suture using Vicryl [Fig. 2 (b)]. Finally, skin closure was performed using subcuticular sutures [Fig. 2 (c)]. At the end of the surgery, which took 1 hour, cystoscopy was performed because of the proximity to the urethra and confirmed no injury to the bladder.

The excised tissue was sent for histopathological examination. The patient remained symptom-free on the first postoperative day and was discharged uneventfully on the second post-operative day. Histopathological examination later confirmed the diagnosis of vulvar endometriosis. During monthly follow-up visits and imaging studies performed every 3 months, physical examination and ultrasound imaging did not show recurrence during the 6-month follow-up period [Fig. 1 (b)]. She never complained of any symptoms related to urinary or sexual function.

## Discussion

Vulvar endometriosis is a rare entity with variable clinical presentations and was first described by Schickele in 1923^[^14^]^. Vulvar endometriosis is most commonly observed following vulvar trauma, although its exact etiology remains uncertain. The mechanisms proposed for the development of endometriosis include endometrial implantation, coelomic metaplasia, lymphatic dissemination, and hematogenous spread^[^1^]^. Among these, direct mechanical implantation appears to be the most plausible hypothesis for explaining scar endometriosis following obstetrical and gynecological procedures^[^9,15–17^]^. The dominant clinical features of vulvar endometriosis include the presence of a painful mass accompanied by cyclic changes^[^18^]^. Surgical excision of the lesion with healthy edges reduces the risk of recurrence^[^19^]^. However, 10.2% of patients have experienced recurrence within a median follow-up duration of 10 months^[^12^]^.

Table 1 shows a summarized description of previously published cases. The literature emphasizes that most patients were of reproductive age with a history of episiotomy following vaginal delivery or prior perineal trauma, supporting the theory of direct implantation of endometrial tissue^[^1,17^]^. The most common chief complaints among patients were cyclic pain, painful perineal swelling, or a palpable vulvar mass, while atypical ulcerative lesions and postmenopausal cases were also reported^[^7^]^.

**Table 1:** Summary of selected previously published cases of perineal and vulvar endometriosis.

First author	Year	Age	History of vaginal delivery	Clinical Presentation	Management
Mishra *et* *al*^[^20^]^	2024	30	Yes	Painful cyclic perineal swelling	Wide local excision with a 1‑cm clear margin combined with GnRH agonist therapy
Sanap *et* *al*^[^21^]^	2024	33	Yes	Painful perineal lump	The nodule was excised with free surgical margins
Tian *et* *al*^[^22^]^	2024	25	No	Cyclic vaginal pain	Surgical excision followed by GnRH agonist therapy
Mehdi *et* *al*^[^23^]^	2023	34	No	Gradually enlarging non‑cyclic painful swelling	Excision with free margins
Feng *et* *al*^[^24^]^	2022	32	Yes	Progressive Cyclic perineal pain and swelling	Complete excision with a 5‑mm healthy tissue margin
Seun-Wallach *et* *al*^[^25^]^	2025	14	No	Painful vulvar ulcer	Surgical excision
Ali *et al*^[^7^]^	2022	40	No	Palpable, firm, semi-mobile vulvar mass	Complete surgical excision
Sarpeitro *et al*^[^26^]^	2023	84	No	Mobile vulvar mass	Complete excision
Nour *et* *al*^[^27^]^	2025	36	Yes	Cyclic pain	Progestin therapy
McComb *et* *al*^[^28^]^	2023	37	Yes	Painful perineal mass	Surgical excision

Previous studies have revealed that the preferred management is complete surgical excision^[^2,29^]^. Vimee Bindra *et al*^[^29^]^ reported a 28-year-old woman with cyclic perineal pain and swelling who had undergone two prior incomplete local excisions, leading to recurrent episiotomy scar endometriosis. Upon re-presentation, she underwent a wide local excision with clear margins, which resulted in complete symptom resolution. In comparison, our patient had a previous incision and drainage procedure that similarly led to recurrence of symptoms, and definitive management in our center was also achieved through wide local excision. Therefore, we suggest that complete surgical excision with clear margins is associated with lower recurrence rates, whereas incomplete procedures may predispose patients to persistent or recurrent disease.

Reporting diverse cases is essential, as it reduces the likelihood of misdiagnosis, inappropriate management, or even neglect of the condition. Compared with previously published cases, our report demonstrates several distinctive features. The lesion was in the upper part of the right labia majora, located away from the episiotomy scar; however, most reported vulvar cases were adjacent to previous episiotomy scars^[^6^]^. Moreover, the lesion caused periurethral deviation, a presentation rarely emphasized in previous reports. These findings complicated both the diagnostic evaluation and surgical planning.

## Conclusion

The key steps for accurate diagnosis and effective management of vulvar endometriosis include obtaining a comprehensive medical and surgical history, careful assessment of the regional anatomy, and selecting an optimal surgical approach, followed by histopathological confirmation. Although vulvar endometriosis is rare, it should be considered in all female patients with a history of vaginal delivery who present with cyclic pain and a palpable perineal mass, particularly during menstruation. The resolution of pain and the improvement in the patient’s quality of life underscore the importance of complete excision of the affected tissue.

## Data Availability

All data used to write this manuscript are available for review by the Editor-in-Chief of this journal on request.

## Research registration unique identifying number (UIN)

IR.TUMS.IKHC.REC.1404.529.
